# Ursodeoxycholic acid treatment did not show protective effect for severe COVID-19 outcomes – a nationwide register study

**DOI:** 10.1186/s12889-026-26908-1

**Published:** 2026-04-11

**Authors:** Huiqi Li, Yiyi Xu, Brian Kirui, Ailiana Santosa, Fredrik Nyberg

**Affiliations:** School of Public Health and Community Medicine, Institute of Medicine, Sahlgrenska Academy, University of Gothenburg, Box 469, Gothenburg, 40530 Sweden

## Abstract

**Background:**

Ursodeoxycholic acid (UDCA), used for treating cholestasis, was reported to protect against severe COVID-19 outcomes. We aimed to assess this potential effect using nationwide Swedish register data.

**Method:**

We included all SARS-CoV-2 test-positive subjects aged ≥ 18 years during 2020–2023, with previous diagnosis of potentially UDCA-treated diseases, including primary biliary cholangitis, liver cirrhosis, autoimmune hepatitis, or cholangitis. Subjects who had filled a UDCA prescription within 6 months before testing positive were considered exposed. Subjects were followed from test-positivity to the earliest of analyzed outcome (COVID-19-related hospitalization, 30-day all-cause mortality, or COVID-19-related death), one year of follow-up, emigration, death, or end of 2023. Control for potential confounding (vaccination status, COVID-19 waves, prior comorbidities, and sociodemographics) was done by adjustment, propensity score weighting (PSW), and doubly robust analysis. Cox regression was used to estimate hazard ratios (HR) with 95% confidence intervals (95%CI).

**Results:**

The study cohort consisted of 833 UDCA-exposed and 3638 non-exposed individuals. The adjusted Cox analysis showed potentially decreased risks (non-significant) among UDCA-exposed for 30-day mortality (HR 0.62, 95%CI 0.34–1.13) and COVID-19 death (0.70, 0.37–1.32), but a smaller non-significant increased risk for hospitalization (1.19, 0.92–1.53). PSW achieved an adequate balance between exposed and non-exposed and showed similar results for 30-day mortality (0.78, 0.40–1.53), COVID-19 death (0.86, 0.43–1.71), and hospitalization (1.15, 0.81–1.61). The doubly robust method showed similar results as PSW.

**Conclusion:**

This study did not provide evidence supporting protective effects for severe COVID-19 outcomes. However, the results are limited by the small cohort of patients treated with UDCA.

**Supplementary Information:**

The online version contains supplementary material available at 10.1186/s12889-026-26908-1.

## Introduction

A previous study [1] indicated that suppressing farnesoid X receptor (FXR) signaling will reduce angiotensin-converting enzyme 2 (*ACE2*) expression and in turn, SARS-CoV-2 infection. One drug targeting this receptor is ursodeoxycholic acid (UDCA), a medication prescribed for treating cholestasis in liver or biliary disease, especially primary biliary cholangitis [2]. Based on experimental evidence supporting this effect, Brevini et al. performed two analyses based on register data. First, in chronic liver disease patients with COVID-19 using registry data that included 31 UDCA users and 155 matched non-users, an association was found between UDCA therapy and better clinical outcomes of COVID-19 (e.g., hospitalization, intensive care unit (ICU) admission and death). Second, among liver transplant recipients with 24 UDCA users and 72 matched non-users, showing that patients on UDCA were less likely to develop moderate or severe COVID-19 outcomes. An earlier study similarly reported lower risk for severe COVID-19 outcomes among 1607 cirrhosis patients on UDCA compared to 1607 propensity score matched (though with quite some unbalanced covariates) patients without UDCA exposure, with the odds ratios (OR) around 0.5 and 95% confidence intervals (95%CI) not covering 1.0 for the 4 outcomes of any COVID-19, symptomatic COVID-19, moderate/severe/critical COVID-19, and severe/critical COVID-19, but not for the outcome of COVID-19-related death [3]. A different study found a protective effect against COVID-19 infection with OR 0.32 (95%CI 0.16–0.64) among 225 patients with chronic liver disease who used UDCA compared to 225 matched patients without UDCA [4]. Additionally, one study of 897 liver transplanted patients from two hospitals identified 326 UDCA users and 571 non-users, and after 1:1 matching, the UDCA group had a lower rate of COVID-19 infection [5].

In contrast, Ojeda-Fernández et al. reported no differences in risk of COVID-19 infection between UDCA users and non-users, in 9617 patients with liver diseases from regional databases in Italy, and no difference regarding moderate or severe COVID-19 outcomes among those infected [6]. Liu and Wang reported very similar proportion of COVID-19 infection in 146 UDCA user and 80 non-user children [7]. Colapietro et al. found similar mortality in COVID-19 hospitalized patients, between the 57 UDCA users and 3790 non-users [8]. The already mentioned study of liver transplanted patients with lower rate of COVID-19 infection in the UDCA group did not find differences regarding severe COVID-19 outcomes [5].

Given the relatively selected and small sample sizes and inconsistent findings on the effect of UDCA on severe COVID-19 outcomes, a population-based study using a nationwide cohort would be beneficial. In this study, we therefore aimed to test if there is any evidence of a protective effect of UDCA against severe COVID-19 outcomes in a nationwide Swedish register database.

## Materials and methods

This is a register-based cohort study using the SCIFI-PEARL (Swedish Covid-19 Investigation for Future Insights – a Population Epidemiology Approach using Register Linkage) project that consists of data for the entire Swedish population from 1 Jan 2015 to present, with multiple registers linked using the Swedish national personal identification number [9]. The data used in this study originate from several registers, including the National Patient Register (NPR, containing inpatient and specialist outpatient medical visits), the National Prescribed Drug Register (NPDR, with drug dispensing records), the National Cause of Death Register (NCDR, with death information), the Swedish Intensive Care Register (SIR, with ICU admission records), the national database of notifiable disease (SmiNet, with positive polymerase chain reaction test for SARS-CoV-2), the National Vaccination Register (NVR, with COVID-19 vaccination records), the Total Population Register (TPR, with basic demographics), and the Longitudinal Integrated Database for Health Insurance and Labour Market Studies (LISA, socioeconomic information). We had data from most of the registers from 2015, except NPDR where we had information from 2018, and SmiNet and NVR from 2020.

### Study population: UDCA use cohort

The main study cohort (referred as “UDCA use cohort”, to distinguish it from other cohorts defined in the sensitivity analysis) consisted of all COVID-19 test-positive adults who had been diagnosed with any of the diagnoses in which UDCA is often used clinically in Sweden (as listed below) up to the index date. We used data up to 31 Dec 2023, and included only individuals who had first positive polymerase chain reaction (PCR) test between 1 Jan 2020 and 31 Oct 2023 (to ensure at least 2 months of follow-up) and with previous diagnosis of potentially UDCA-treated diseases recorded using International Classification of Diseases, 10th revision (ICD-10) codes, including primary biliary cholangitis (PBC, ICD-10 code K74.3), cirrhosis of liver (K74.6), cholangitis (K83.0), or autoimmune hepatitis (K75.4), identified from the NPR. Based on a conservative assumption of minimum incubation time, the index date was set as two days before test-positivity date. Individuals under 18 years of age ate the index date were excluded. Individuals were followed from the index date until event dates (see outcomes below) or censor dates (including at one year of follow-up, emigration, death, or 31 Dec 2023).

### Exposure

The exposure of the study is if there was dispensed UDCA prescriptions during the six months prior to the index date, which were obtained using the NPDR. Individuals with dispensing of UDCA (Anatomical Therapeutic Chemical [ATC] code A05AA02) during the period were categorized as exposed and this exposure was kept as time-fixed.

### Outcomes

Four COVID-19 outcomes were defined:

1. Hospitalization related to COVID-19 was identified using NPR, as an inpatient care record with the diagnosis of COVID-19 (ICD-10 codes U07.1 or U07.2) as primary or secondary diagnosis. The first admission date for each person was considered the date of the event.
2. Admission to ICU related to COVID-19 was identified similarly as above, using data from SIR.
3. 30-day mortality after test-positivity was identified as death of any cause within 30 days from test-positivity using data from NCDR.
4. COVID-19-related death was identified similarly using data from NCDR where U07.1 or U07.2 were registered as underlying or contributing cause of death, during the full follow-up.

### Covariates

Demographic and socioeconomic information collected prior to 2020 (as a surrogate for similar information on the index date) was obtained from TPR and LISA. Education level was grouped into primary, upper secondary, tertiary, and unknown; disposable income per consumption unit for family was categorized into quartiles plus unknown; birth country was grouped according to the World Bank classification [10], except that Sweden was considered as a separate category. COVID-19 vaccination status was assessed as the number of vaccination doses received before index date, using data from NVR. COVID-19 variant waves at the time of positive test were defined based on dominant virus variant during calendar period: wave 1 (1 Jan 2020 to 31 Jan 2021) - wildtype and/or mixed variants of virus; wave 2 (1 Feb 2021 to 30 Jun 2021) - Alpha variant; wave 3 (1 Jul 2021 to 31 Dec 2021) - Delta variant; wave 4 (1 Jan 2022 onwards) - Omicron variant. Disease history from 2015 to the index date was assessed for the following: cardiovascular (ICD-10: I00-I99), kidney (N00-N19, I12, I13.1, I13.2), psychiatric (F20-F39), respiratory (J00-J99), and liver (K70-K77) diseases, diabetes (E10-E14) and dementia (F00-F03, F051, G30, G31.1) were assessed.

Liver transplant patients were identified using the ICD-10 code Z94.4 and KVÅ (Swedish classification of procedures) codes JJC00, JJC96, JJC10, JJC20, and among them only those under immunosuppressor treatment (ATC: L04AD01, L04AD02, L04AA06, L04AA10, L04AA18, L04AX03, L04AX01, H02AB06) were considered in the analyses.

### Statistical methods

Demographic and socioeconomic characteristics were described in the exposed and non-exposed group separately, where the mean and standard deviation of age on index date, and count and percentage for all other variables were presented.

Three analyses were performed to estimate hazard ratios (HR) and 95%CI for each outcome.

1. Adjusted Cox: Cox regression with all covariates included in the models for adjustment, where age was adjusted using a restricted cubic spline with 5 knots based on Harrell’s recommended percentiles [11].
2. Propensity score weighting (PSW) analysis: The propensity score (ps) was estimated using a gradient boosted model including all the covariates listed in Table 1, and a predicted probability of UDCA exposure was obtained for each individual. Standardized morbidity ratio (SMR) weights were generated, i.e. weights set to 1 for the UDCA-exposed, and to ps/(1-ps) for the non-exposed [12]. Cox regressions with the weights were used to obtain effect estimates.
3. Doubly robust: Cox regressions with the weights from PSW, plus including all covariates for adjustment (age as restricted cubic spline).

All analyses were performed using Stata 17 (StataCorp LLC) and R (4.0.2, R Core Team). Gradient boosted model for ps estimation was performed using the R package *twang* v2.5 [13].

### Sensitivity analysis

To explore the effect in different target populations, we defined three additional sensitivity analysis cohorts and performed the same statistical analysis in each cohort. All cohorts were defined using the same data source as in the main analysis, with differences regarding inclusion criteria as:

1. Full population cohort – in order to assess the effect in the general population. This consisted of COVID-19 test-positive adults from 1 Jan 2020 to 31 Oct 2023.
2. PBC cohort – for a stricter indication. This consisted of COVID-19 test-positive adults who had had received a diagnosis of PBC, the approved indication for UDCA prescription in Sweden, from 1 Jan 2015 to index date.
3. Liver transplant cohort – for replicating the previous study [1]. This consisted of COVID-19 test-positive adults with a liver transplant before index and who were still under immunosuppressor medication on index date.

## Results

### Characteristics of the main UDCA use cohort before and after PSW

**Fig. 1:**
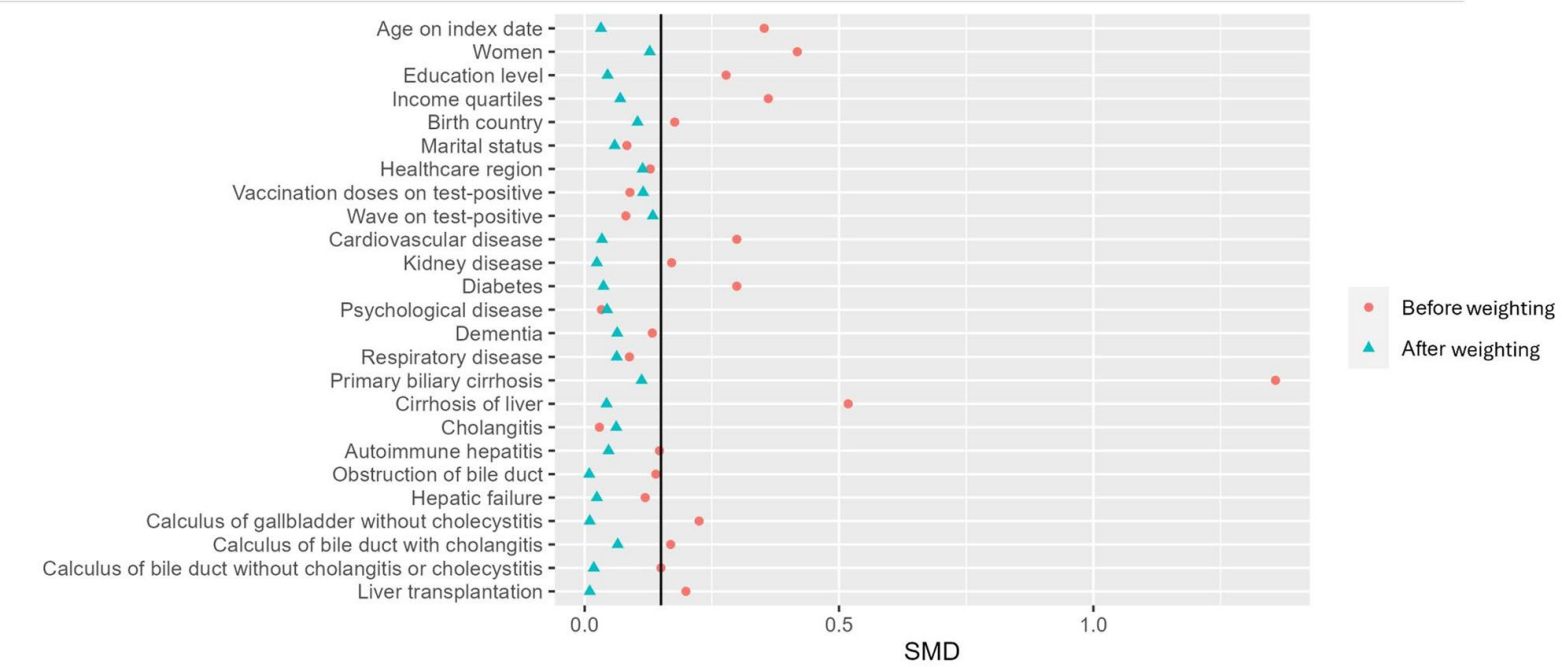
Standardized mean differences (SMD) in covariates before and after propensity score weighting in the ursodeoxycholic acid (UDCA) exposed and non-exposed groups in the UDCA use cohort, among patients with a positive COVID-19 test in the Swedish population 1 Jan 2020 to 31 Dec 2023. UDCA use cohort consisted of all COVID-19 test-positive adults who had received any of the diagnoses in which UDCA is often used, i.e., a diagnosis of primary biliary cholangitis (PBC, International Classification of Diseases, 10th revision (ICD-10) code K74.3), cirrhosis of liver (K74.6), cholangitis (K83.0), and autoimmune hepatitis (K75.4), from 1 Jan 2015 to the date of each individual’s first positive test for SARS-CoV-2. The vertical black line represents SMD = 0.15, values below it (dots on the left side of the line) represents good balance between groups

The 833 exposed and 3 638 unexposed individuals were similar in many characteristics, but the exposed group was slightly younger, with higher proportion of women, fewer individuals with low income, and a lower prevalence of cardiovascular disease and diabetes (Table 1). Additionally, the proportions of individuals with different liver diseases were also imbalanced before weighting. However, after weighting, most covariates were much better balanced, with all standardized mean differences below 0.15 [14] (Fig. 1).

**Table 1:** Demographic and socioeconomic characteristics and comorbidities of the ursodeoxycholic acid (UDCA) exposed and non-exposed group in the UDCA use cohort, among patients with a positive COVID-19 test in the Swedish population 1 Jan 2020 to 31 Dec 2023

Use Cohort	Exposed	Non-Exposed		SMD*	
		Before weighting	After weighting	Before weighting	After weighting
Count, N	833	3638			
Age on test-positive, mean (SD)	54.7 ± 16.9	60.9 ± 18.2	54.2 ± 16.5	0.353	0.032
Women, N (%)	573 (68.8%)	1769 (48.6%)	428.2 (62.7%)	0.418	0.128
Education level, N (%)				0.278	0.045
primary	126 (15.1%)	849 (23.3%)	101.0 (14.8%)		
upper secondary	360 (43.2%)	1650 (45.4%)	310.0 (45.4%)		
tertiary	335 (40.2%)	1057 (29.1%)	261.8 (38.4%)		
unknown	12 (1.4%)	82 (2.3%)	9.8 (1.4%)		
Income quartiles, N (%)				0.361	0.070
low	188 (22.6%)	1399 (38.5%)	162.7 (23.8%)		
lower-middle	195 (23.4%)	787 (21.6%)	144.6 (21.2%)		
upper-middle	215 (25.8%)	694 (19.1%)	170.8 (25.0%)		
high	234 (28.1%)	755 (20.8%)	204.1 (29.9%)		
unknown	1 (0.1%)	3 (0.1%)	0.3 (0.0%)		
Birth country, N (%)				0.177	0.104
Sweden	694 (83.3%)	2807 (77.2%)	571.5 (83.7%)		
high income	45 (5.4%)	278 (7.6%)	30.5 (4.5%)		
low income	20 (2.4%)	140 (3.8%)	24.0 (3.5%)		
lower-middle income	13 (1.6%)	114 (3.1%)	13.4 (2.0%)		
upper-middle income	53 (6.4%)	252 (6.9%)	34.5 (5.1%)		
unknown	8 (1.0%)	47 (1.3%)	8.6 (1.3%)		
Marital status, N (%)				0.083	0.059
married	396 (47.5%)	1581 (43.5%)	306.2 (44.9%)		
not-married	436 (52.3%)	2054 (56.5%)	376.0 (55.1%)		
unknown	1 (0.1%)	3 (0.1%)	0.3 (0.0%)		
Healthcare region, N (%)				0.129	0.114
North	70 (8.4%)	268 (7.4%)	63.7 (9.3%)		
Stockholm	214 (25.7%)	981 (27.0%)	174.4 (25.6%)		
Southeast	92 (11.0%)	406 (11.2%)	70.4 (10.3%)		
South	138 (16.6%)	639 (17.6%)	137.8 (20.2%)		
Uppsala-Örebro	196 (23.5%)	701 (19.3%)	144.8 (21.2%)		
West	122 (14.6%)	640 (17.6%)	90.9 (13.3%)		
unknown	1 (0.1%)	3 (0.1%)	0.3 (0.0%)		
Vaccination doses before positive COVID-19 test, N (%)				0.089	0.115
0 dose	395 (47.4%)	1746 (48.0%)	304.1 (44.6%)		
1 dose	21 (2.5%)	143 (3.9%)	30.3 (4.4%)		
2 doses	165 (19.8%)	655 (18.0%)	143.7 (21.0%)		
3 doses or more	252 (30.3%)	1094 (30.1%)	204.5 (30.0%)		
Infection wave when tested positive, N (%)				0.081	0.134
Wave 1 (Jan 2020 – Jan 2021)	236 (28.3%)	1115 (30.6%)	175.6 (25.7%)		
Wave 2 (Feb 2021 – Jun 2021)	152 (18.2%)	564 (15.5%)	113.9 (16.7%)		
Wave 3 (Jul 2021 – Dec 2021)	70 (8.4%)	293 (8.1%)	83.2 (12.2%)		
Wave 4 (Jan 2022 – Jun 2022)	375 (45.0%)	1666 (45.8%)	309.7 (45.4%)		
Cardiovascular disease, N (%)	296 (35.5%)	1825 (50.2%)	231.5 (33.9%)	0.299	0.034
Kidney disease, N (%)	66 (7.9%)	478 (13.1%)	58.6 (8.6%)	0.171	0.024
Diabetes, N (%)	93 (11.2%)	807 (22.2%)	84.4 (12.4%)	0.299	0.037
Psychological disease, N (%)	50 (6.0%)	248 (6.8%)	34.1 (5.0%)	0.033	0.044
Dementia, N (%)	8 (1.0%)	100 (2.7%)	11.6 (1.7%)	0.133	0.064
Respiratory disease, N (%)	197 (23.6%)	1000 (27.5%)	180.1 (26.4%)	0.088	0.063
Primary biliary cholangitis (K74.3), N (%)	443 (53.2%)	100 (2.7%)	324.9 (47.6%)	1.358	0.112
Cirrhosis of liver (K74.6), N (%)	131 (15.7%)	1381 (38.0%)	118.3 (17.3%)	0.518	0.043
Cholangitis (K83.0), N (%)	369 (44.3%)	1664 (45.7%)	323.3 (47.4%)	0.029	0.062
Autoimmune hepatitis (K75.4), N (%)	126 (15.1%)	755 (20.8%)	92.0 (13.5%)	0.147	0.047
Obstruction of bile duct (K83.1), N (%)	79 (9.5%)	210 (5.8%)	66.5 (9.7%)	0.140	0.009
Hepatic failure (K72), N (%)	39 (4.7%)	274 (7.5%)	28.6 (4.2%)	0.119	0.024
Calculus of gallbladder without cholecystitis (K80.2), N (%)	38 (4.6%)	380 (10.4%)	29.7 (4.4%)	0.225	0.010
Calculus of bile duct with cholangitis (K80.3), N (%)	20 (2.4%)	208 (5.7%)	23.8 (3.5%)	0.169	0.065
Calculus of bile duct without cholangitis or cholecystitis (K80.5), N (%)	26 (3.1%)	229 (6.3%)	23.5 (3.4%)	0.150	0.018
Liver transplant, N (%)	94 (11.3%)	209 (5.7%)	79.2 (11.6%)	0.199	0.010

*SMD: standardized mean difference, between exposed group and non-exposed group

### COVID-19 related outcomes

**Fig. 2:**
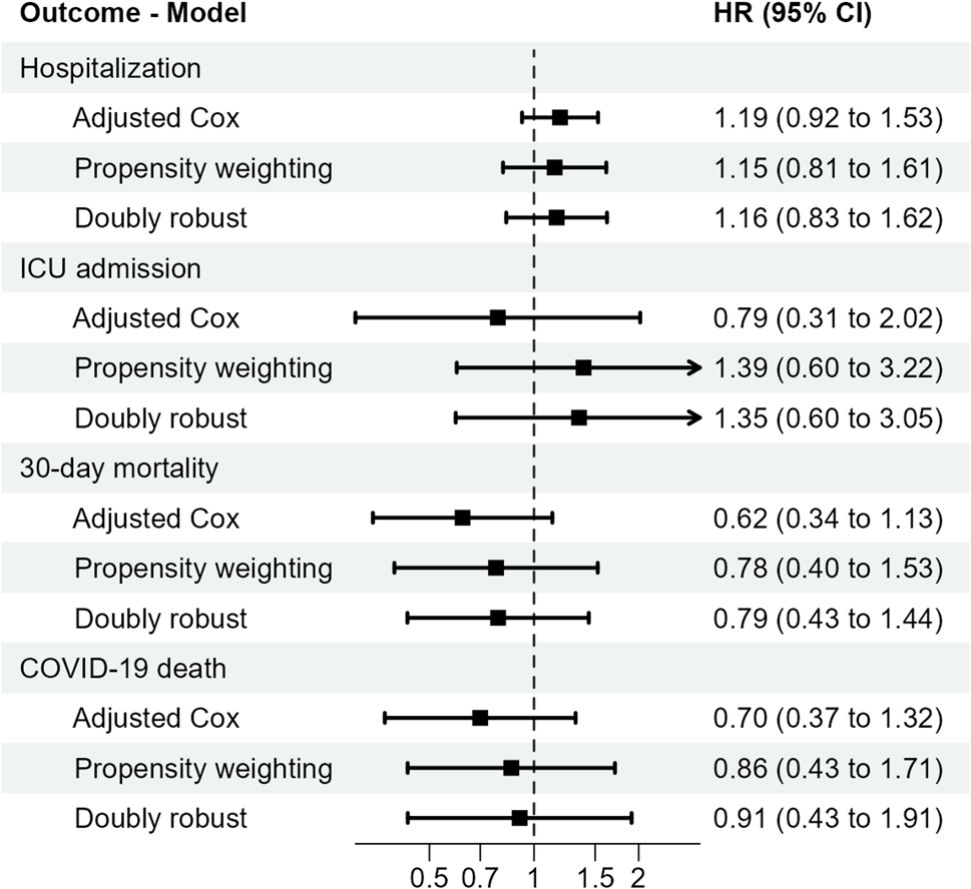
Adjusted hazard ratios and 95% confidence intervals of four COVID-19 outcomes comparing ursodeoxycholic acid (UDCA) exposed to non-exposed group in the UDCA use cohort, by 3 different analytical methods. UDCA use cohort consisted of all COVID-19 test-positive adults who had received any of the diagnoses in which UDCA is often used, i.e., a diagnosis of primary biliary cholangitis (PBC, International Classification of Diseases, 10th revision (ICD-10) code K74.3), cirrhosis of liver (K74.6), cholangitis (K83.0), and autoimmune hepatitis (K75.4), from 1 Jan 2015 to the date of each individual’s first positive test for SARS-CoV-2

Hazard ratios (HR) and 95% confidence intervals (95%CI) for all 4 outcomes are presented in Fig. 2.

The three methods mostly showed the same direction of effect: the UDCA-exposed group had a slightly increased HR for hospitalization but a decreased HR for 30-day overall mortality and COVID-19 death, though none of these results were statistically significant. However, the results for ICU admission were inconsistent. The adjusted Cox model showed a decreased while the other two methods showed an increased HR (also not statistically significant) for the UDCA-exposed group. It is important to note that the number of outcome events and power was more limited, which reduced the statistical power for these analyses.

### Sensitivity analyses

The three additional cohorts varied in terms of sample size and balance. The full population cohort had the largest sample size, with 1 185 individuals in the UDCA-exposed and 1 985 671 in the non-exposed group, but it was far from equipoise. Even after weighting, it remained imbalanced, with three covariates showing SMD > 0.15 (Table S1, Figure S1).The PBC cohort, while the most relevant to UDCA “on indication” prescription, was highly restricted, resulting in very small sample size and a high prevalence of UDCA use, with 443 in the UDCA-exposed and 100 in non-exposed groups (Table S2, Figure S2). Similarly, the liver transplant cohort was also very restricted, with 114 and 397 individuals in the exposed and non-exposed groups, respectively (Table S3, Figure S3). Due to the small size of non-exposed groups in both PBC and liver transplant cohorts, neither cohort achieved good balance after weighting either, with more than five covariates having SMD > 0.15.

Despite the issue of poor balance, the results from these three cohorts were mostly in line with the main study cohort, showing a slightly increased risk for hospitalization and a decreased risk for mortality (Figure S4). However, due to the very small sample sizes, certain analyses in the PBC cohort and the liver transplant cohort either did not converge or produced extremely wide 95%CIs.

## Discussion

This study assesses severe COVID-19 outcomes related to exposure to the drug UDCA and provides weak evidence in support of a potential protective effect regarding mortality, despite a very weak suggestion of an increased risk of COVID-19-related hospitalization.

Lower risks of mortality and cause-specific death were found in the UDCA-exposed group; however, the results were not statistically significant, even within the nationwide study population. The direction of decreased risk of mortality and death could be explained by the mechanism introduced by the paper by Brevini et al., that UDCA by suppressing farnesoid X receptor (FXR) signaling could reduce *ACE2* expression and therefore lead to a better clinical outcome of COVID-19 [1]. Several other studies have also shown decreased risk for severe COVID-19 outcomes in various study populations [3–5]. The results from this study add some additional, though rather weak, evidence on this topic.

Though in this study we focused on the adult population, it is of interest that earlier studies using pediatric data from a large tertiary liver transplantation center suggested that UDCA exposure was not associated with differences in COVID-19-related outcomes among children with chronic liver disease, who generally experienced a favorable clinical course [15, 16].

On the other hand, a suggested increased risk of hospitalization in relation to COVID-19 was observed in the UDCA-exposed group. We defined this outcome based on hospitalization records with COVID-19 diagnosis as primary or secondary diagnosis, and additionally performed an analysis considering only the primary diagnosis was considered, which provided similar results (data not shown). Therefore, the increased risk of hospitalization indicates to some extent that the patients undergoing UDCA treatment may be more likely require inpatient care for COVID-19. There could be many plausible reasons: 1) the UDCA users had increased risk for severe COVID-19 which requires inpatient care; 2) the UDCA users were more prioritized for inpatient care when COVID-19 infection was observed; 3) there were certain interactions between COVID-19 infection and the liver comorbidities that would lead to inpatient admission. It is unclear from our study what were the dominating reasons.

Based on the results of this study, the evidence is not strong enough to recommend UDCA as a protective treatment for the general population or people with liver disease. Instead, prevention of adverse COVID-19 outcomes in patients with liver diseases relies primarily on established evidence-based strategies including SARS-CoV-2 vaccination [17], early initiation of antiviral therapy in high-risk individuals, optimization of metabolic comorbidities, and careful monitoring of patients with advanced fibrosis or cirrhosis [18]. Maintenance of standard-of-care management for underlying liver disease is also essential to reduce vulnerability to severe infection [19].

This study performed a current-users versus non-users comparison, while we are consciously aware that the design is flawed for causal inference [20]. The reason of the design was for the consideration of sample size. The results found in this study cannot be translated to the effect of giving UDCA to people, which should be investigated by comparing new-users versus non-users. Considering this drawback, together with the opposite direction found in this study regarding hospitalization and mortality and death, our results are not strong enough to recommend the use of UDCA as a prevention measure against severe COVID-19 outcomes.

UDCA treatment is generally prescribed to a specific target group with liver and biliary diseases, such as PBC, with a certain level of disease severity. In our study, there were 543 individuals with PBC (443 treated with UDCA and 100 not treated with UDCA), with PBC diagnosis identified from medical care records 2015 to index date, and the UDCA prescription identified from drug dispensing records six months preceding the index date. Consequently, individuals with a recorded PBC diagnosis but without documented UDCA treatment at the index date may be explained by several factors, including recent diagnosis with treatment initiation occurring after the index date, diagnostic uncertainty related to registry-based case identification (e.g., coding errors or subsequent reclassification), UDCA intolerance or treatment discontinuation, advanced disease with limited expected benefit, and incomplete capture of prescriptions outside the defined dispensing window.

Additionally, confounding by indication is a likely issue when comparing UDCA-exposed individuals with the general population or, to some extent, with any unexposed group. To address this, we applied multiple cohort definitions as well as multiple approaches to adjusting for potential confounding due to group differences. The full population cohort may be too broad and imbalanced, even after adjustment or PSW analyses. The PBC cohort, on the other hand, is too restricted, with small sample size and with common UDCA exposure; similarly, the liver transplant cohort also has a small sample size. Consequently, the latter two cohorts had poor balance even after adjustments or PSW. In our opinion, the main study cohort - the UDCA use cohort - is the most appropriate for investigating the research question, as it includes patients with a spectrum of conditions (either with registered indication or broad off-label use) with a reasonable clinical equipoise, and it showed relatively good balance after PSW. The cohort selection was partly data-driven, with the inclusion diagnoses based on a priori observations to identify common conditions of individuals treated with UDCA.

We used several analytic methods to assess the association, but it is important to note that the adjusted Cox and PSW based methods reflect different estimands [12]. The adjusted Cox model estimates the average treatment effect on everyone (ATE), while PSW with SMR weighting estimates the average treatment effect on the treated (ATT). These two estimates are numerically the same only if there is no effect modification for the exposure (UDCA in this case) by any of the covariates, but such effect modification may often exist. In this study, the PSW and doubly robust results were more conservative (closer to null) than the adjusted Cox, potentially reflecting the difference between ATT and ATE, or more comprehensive confounding control with the advanced methods.

A key strength of the study is the use of comprehensive nationwide population data with a large sample size, as well as the good quality of data from Swedish registers, which not only provided the basis of a sound study, but also enabled the different study settings used in the sensitivity analysis. While unmeasured confounding is often a concern in register-based studies, we had access to extensive data covering various aspects of demographics, socioeconomics, and medical history. This wide range of information enabled us to control for many relevant factors – more than other similar register-based studies – and to achieve reasonably good balance in the PS analyses. Therefore, strong unmeasured residual confounding is a less likely explanation for our results. However, the potential possibility of residual confounding cannot be fully excluded, and randomized controlled trials are needed to fully address this issue. Other limitations to acknowledge include: UDCA exposure status was based on pharmacy dispensing records, which may not perfectly reflect actual usage. In this study, the most likely scenario of exposure misclassification is that the individuals who dispensed UDCA did not adhere to the treatment because of various reasons including change of symptoms or simply forgot to intake the medicine. This potential misclassification would bias the results towards null, which may partly explain that we did not observe strong evidence. It would be ideal to obtain patient-reported data on drug adherence, but given the nature of register-based study, it was not feasible since we could not trace individuals in the study. Nevertheless, given that UDCA is prescribed for specific disease conditions, we can assume fairly good adherence to the treatment. It should be noted that low event numbers for critical outcomes (e.g., ICU admission) and small sample sizes in restricted sensitivity analysis cohorts (e.g., PBC, liver transplant patients) may limit result reliability. However, it was not possible to expand the sample size further as it was already based on nation-wide data.

## Conclusions

In conclusion, this nationwide register-based study of the Swedish population did not provide clear evidence suggesting UDCA-exposed individuals had decreased risk of severe COVID-19 outcomes. Point estimates suggest a possible weak decrease for COVID-19-related mortality or death, similar to prior studies. However, the results are limited by the small cohort of patients treated with UDCA and the potential effect is not strong enough to recommend UDCA as a preventive measure against severe COVID-19 outcomes.

## Supplementary Information

Supplementary Material 1.

## Data Availability

The data in this study are deidentified individual-level data from Swedish healthcare registers and can be obtained from the respective Swedish public data holders on the basis of ethics approval for the research in question, subject to relevant legislation, processes, and data protection.
