# Supplementary material for: Ursodeoxycholic acid treatment did not show protective effect for severe COVID-19 outcomes – a nationwide register study
Source: BMC Public Health. 2026 Apr 11;26:1240. doi: 10.1186/s12889-026-26908-1 (PMC13085402; doi:10.1186/s12889-026-26908-1)
Supplement: Supplementary file 1 — Supplementary Material 1. [file 12889_2026_26908_MOESM1_ESM.docx]

Table S1. Demographic and socioeconomic characteristics and comorbidities of the ursodeoxycholic acid (UDCA) exposed and non-exposed group in the full population cohort, among patients with a positive COVID-19 test in the Swedish population 1 Jan 2020 to 31 Dec 2023.

| Full population cohort | Exposed | Non-Exposed | | SMD* | |
| --- | --- | --- | --- | --- | --- |
|  |  | Before weighting | After weighting | Before weighting | After weighting |
| Count, N | 1185 | 1985671 |  |  |  |
| Age on test-positive, mean (SD) | 52.18 (17.65) | 43.93 (16.52) | 50.35 (17.65) | 0.483 | 0.104 |
| Women, N (%) | 826 (69.7) | 1061940 (53.5) | 784.3 (60.3) | 0.338 | 0.197 |
| Education level, N (%) |  |  |  | 0.030 | 0.124 |
| primary | 172 (14.5) | 301433 (15.2) | 182.4 (14.0) |  |  |
| upper-secondary | 499 (42.1) | 836164 (42.1) | 620.9 (47.8) |  |  |
| tertiary | 486 (41.0) | 795030 (40.0) | 475.7 (36.6) |  |  |
| unknown | 28 (2.4) | 53044 (2.7) | 20.8 (1.6) |  |  |
| Income quartiles, N (%) |  |  |  | 0.084 | 0.149 |
| low | 292 (24.6) | 481483 (24.2) | 402.3 (31.0) |  |  |
| lower-middle | 271 (22.9) | 492732 (24.8) | 273.2 (21.0) |  |  |
| upper-middle | 295 (24.9) | 497666 (25.1) | 316.4 (24.3) |  |  |
| high | 323 (27.3) | 498199 (25.1) | 303.8 (23.4) |  |  |
| unknown | 4 (0.3) | 15591 (0.8) | 4.0 (0.3) |  |  |
| Birth country, N (%) |  |  |  | 0.132 | 0.135 |
| Sweden | 949 (80.1) | 1519960 (76.5) | 1014.2 (78.0) |  |  |
| high income | 75 (6.3) | 111926 (5.6) | 64.5 (5.0) |  |  |
| low income | 34 (2.9) | 94308 (4.7) | 60.3 (4.6) |  |  |
| lower-middle income | 30 (2.5) | 67950 (3.4) | 38.6 (3.0) |  |  |
| upper-middle income | 82 (6.9) | 156062 (7.9) | 93.3 (7.2) |  |  |
| unknown | 15 (1.3) | 35465 (1.8) | 28.9 (2.2) |  |  |
| Marital status, N (%) |  |  |  | 0.147 | 0.189 |
| married | 558 (47.1) | 799591 (40.3) | 491.3 (37.8) |  |  |
| not-married | 623 (52.6) | 1170509 (58.9) | 804.5 (61.9) |  |  |
| unknown | 4 (0.3) | 15571 (0.8) | 4.0 (0.3) |  |  |
| Healthcare region, N (%) |  |  |  | 0.109 | 0.093 |
| North | 89 (7.5) | 146898 (7.4) | 107.6 (8.3) |  |  |
| Stockholm | 300 (25.3) | 475889 (24.0) | 294.3 (22.6) |  |  |
| Southeast | 125 (10.5) | 193317 (9.7) | 142.3 (10.9) |  |  |
| South | 209 (17.6) | 364777 (18.4) | 254.7 (19.6) |  |  |
| Uppsala-Örebro | 264 (22.3) | 405121 (20.4) | 265.8 (20.5) |  |  |
| West | 194 (16.4) | 384098 (19.3) | 231.0 (17.8) |  |  |
| unknown | 4 (0.3) | 15571 (0.8) | 4.0 (0.3) |  |  |
| Vaccination doses on test-positive, N (%) |  |  |  | 0.353 | 0.202 |
| 0 dose | 577 (48.7) | 1063554 (53.6) | 600.8 (46.2) |  |  |
| 1 dose | 38 (3.2) | 62310 (3.1) | 55.5 (4.3) |  |  |
| 2 doses | 243 (20.5) | 578324 (29.1) | 361.3 (27.8) |  |  |
| 3 doses or more | 327 (27.6) | 281483 (14.2) | 282.1 (21.7) |  |  |
| Wave on test-positive, N (%) |  |  |  | 0.122 | 0.122 |
| Wave 1 (Jan 2020 – Jan 2021) | 343 (28.9) | 522448 (26.3) | 331.3 (25.5) |  |  |
| Wave 2 (Feb 2021 – Jun 2021) | 206 (17.4) | 423057 (21.3) | 247.0 (19.0) |  |  |
| Wave 3 (Jul 2021 – Dec 2021) | 97 (8.2) | 192659 (9.7) | 144.3 (11.1) |  |  |
| Wave 4 (Jan 2022 – Jun 2022) | 539 (45.5) | 847507 (42.7) | 577.1 (44.4) |  |  |
|  |  |  |  |  |  |
| Cardiovascular disease, N (%) | 376 (31.7) | 194644 (9.8) | 341.4 (26.3) | 0.561 | 0.121 |
| Kidney disease, N (%) | 94 (7.9) | 34814 (1.8) | 96.8 (7.4) | 0.291 | 0.018 |
| Diabetes, N (%) | 127 (10.7) | 54994 (2.8) | 122.3 (9.4) | 0.321 | 0.043 |
| Psychological disease, N (%) | 67 (5.7) | 76959 (3.9) | 72.0 (5.5) | 0.084 | 0.005 |
| Dementia, N (%) | 9 (0.8) | 13566 (0.7) | 21.4 (1.7) | 0.009 | 0.082 |
| Respiratory disease, N (%) | 281 (23.7) | 192948 (9.7) | 304.6 (23.4) | 0.382 | 0.007 |
| Primary biliary cirrhosis (K74.3), N (%) | 443 (37.4) | 100 (0.0) | 517.3 (39.8) | 1.092 | 0.05 |
| Cirrhosis of liver (K74.6), N (%) | 131 (11.1) | 1381 (0.1) | 135.4 (10.4) | 0.494 | 0.02 |
| Cholangitis (K83.0), N (%) | 369 (31.1) | 1664 (0.1) | 404.5 (31.1) | 0.947 | <0.001 |
| Autoimmune hepatitis (K75.4), N (%) | 126 (10.6) | 755 (0.0) | 143.4 (11.0) | 0.485 | 0.013 |
| Obstruction of bile duct (K83.1), N (%) | 87 (7.3) | 666 (0.0) | 91.5 (7.0) | 0.395 | 0.012 |
| Hepatic failure (K72), N (%) | 43 (3.6) | 879 (0.0) | 47.7 (3.7) | 0.269 | 0.002 |
| Calculus of gallbladder  without cholecystitis (K80.2), N (%) | 63 (5.3) | 24389 (1.2) | 44.9 (3.5) | 0.231 | 0.091 |
| Calculus of bile duct with  cholangitis (K80.3), N (%) | 23 (1.9) | 803 (0.0) | 27.6 (2.1) | 0.193 | 0.013 |
| Calculus of bile duct without cholangitis  or cholecystitis (K80.5), N (%) | 42 (3.5) | 3367 (0.2) | 24.2 (1.9) | 0.252 | 0.104 |
| Liver transplantation, N (%) | 114 (9.6) | 397 (0.0) | 148.0 (11.4) | 0.460 | 0.058 |

*SMD: standardized mean difference, between exposed group and non-exposed group.


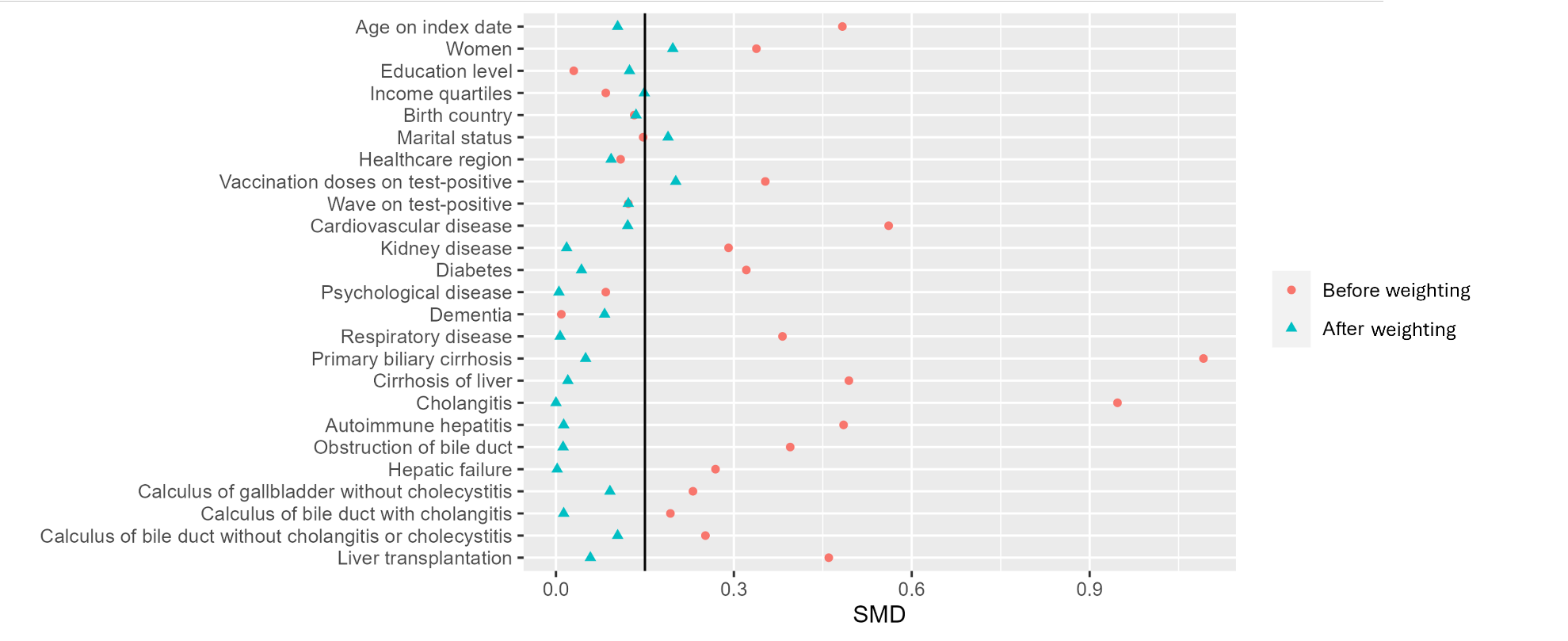


Figure S1. Standardized mean differences (SMD) in covariates before and after propensity score weighting in the ursodeoxycholic acid (UDCA) exposed and non-exposed groups in the full population cohort, among patients with a positive COVID-19 test in the Swedish population 1 Jan 2020 to 31 Dec 202 3. The full population cohort consisted of all COVID-19 test-positive adults. The vertical black line represents SMD=0.15, values below it (dots on the left side of the line) represents good balance between groups.

Table S2. Demographic and socioeconomic characteristics and comorbidities of the ursodeoxycholic acid (UDCA) exposed and non-exposed group in the PBC (primary biliary cirrhosis) cohort, among patients with a positive COVID-19 test in the Swedish population 1 Jan 2020 to 31 Dec 2023.

| PBC cohort | Exposed | Non-Exposed | | SMD* | |
| --- | --- | --- | --- | --- | --- |
|  |  | Before weighting | After weighting | Before weighting | After weighting |
| Count, N | 443 | 100 | 312.83 |  |  |
| Age on test-positive, mean (SD) | 61.48 (13.62) | 59.75 (15.91) | 62.70 (12.34) | 0.117 | 0.094 |
| Women, N (%) | 392 (88.5) | 81 (81.0) | 277.1 (88.6) | 0.209 | 0.003 |
| Education level, N (%) |  |  |  | 0.114 | 0.159 |
| primary | 78 (17.6) | 16 (16.0) | 68.6 (21.9) |  |  |
| upper-secondary | 198 (44.7) | 50 (50.0) | 144.8 (46.3) |  |  |
| tertiary | 160 (36.1) | 33 (33.0) | 92.5 (29.6) |  |  |
| unknown | 7 (1.6) | 1 (1.0) | 7.0 (2.2) |  |  |
| Income quartiles, N (%) |  |  |  | 0.38 | 0.097 |
| low | 112 (25.3) | 43 (43.0) | 89.3 (28.5) |  |  |
| lower-middle | 105 (23.7) | 18 (18.0) | 67.2 (21.5) |  |  |
| upper-middle | 98 (22.1) | 17 (17.0) | 61.9 (19.8) |  |  |
| high | 128 (28.9) | 22 (22.0) | 94.4 (30.2) |  |  |
| unknown |  |  |  |  |  |
| Birth country, N (%) |  |  |  | 0.31 | 0.256 |
| Sweden | 350 (79.0) | 73 (73.0) | 243.9 (78.0) |  |  |
| high income | 33 (7.4) | 7 (7.0) | 19.0 (6.1) |  |  |
| low income | 10 (2.3) | 7 (7.0) | 16.1 (5.2) |  |  |
| lower-middle income | 6 (1.4) | 3 (3.0) | 5.3 (1.7) |  |  |
| upper-middle income | 40 (9.0) | 7 (7.0) | 18.5 (5.9) |  |  |
| unknown | 4 (0.9) | 3 (3.0) | 9.9 (3.2) |  |  |
| Marital status, N (%) |  |  |  | 0.283 | 0.021 |
| married | 230 (51.9) | 38 (38.0) | 159.3 (50.9) |  |  |
| not-married | 213 (48.1) | 62 (62.0) | 153.7 (49.1) |  |  |
| unknown | 0 (0) | 0 (0) | 0 (0) |  |  |
| Healthcare region, N (%) |  |  |  | 0.221 | 0.181 |
| North | 39 (8.8) | 11 (11.0) | 29.7 (9.5) |  |  |
| Stockholm | 106 (23.9) | 22 (22.0) | 70.1 (22.4) |  |  |
| Southeast | 44 (9.9) | 12 (12.0) | 28.8 (9.2) |  |  |
| South | 84 (19.0) | 25 (25.0) | 78.5 (25.1) |  |  |
| Uppsala-Örebro | 98 (22.1) | 18 (18.0) | 68.5 (21.9) |  |  |
| West | 72 (16.3) | 12 (12.0) | 37.3 (11.9) |  |  |
| unknown | 0 (0) | 0 (0) | 0 (0) |  |  |
| Vaccination doses on test-positive, N (%) |  |  |  | 0.206 | 0.068 |
| 0 dose | 218 (49.2) | 43 (43.0) | 151.0 (48.3) |  |  |
| 1 dose | 12 (2.7) | 5 (5.0) | 12.1 (3.9) |  |  |
| 2 doses | 75 (16.9) | 23 (23.0) | 51.0 (16.3) |  |  |
| 3 doses or more | 138 (31.2) | 29 (29.0) | 98.7 (31.5) |  |  |
| Wave on test-positive, N (%) |  |  |  | 0.254 | 0.142 |
| Wave 1 (Jan 2020 – Jan 2021) | 134 (30.2) | 27 (27.0) | 87.3 (27.9) |  |  |
| Wave 2 (Feb 2021 – Jun 2021) | 82 (18.5) | 14 (14.0) | 56.0 (17.9) |  |  |
| Wave 3 (Jul 2021 – Dec 2021) | 34 (7.7) | 15 (15.0) | 36.9 (11.8) |  |  |
| Wave 4 (Jan 2022 – Jun 2022) | 193 (43.6) | 44 (44.0) | 132.6 (42.4) |  |  |
|  |  |  |  |  |  |
| Cardiovascular disease, N (%) | 168 (37.9) | 43 (43.0) | 103.8 (33.2) | 0.104 | 0.099 |
| Kidney disease, N (%) | 36 (8.1) | 14 (14.0) | 29.2 (9.3) | 0.188 | 0.043 |
| Diabetes, N (%) | 45 (10.2) | 12 (12.0) | 28.6 (9.2) | 0.059 | 0.034 |
| Psychological disease, N (%) | 30 (6.8) | 6 (6.0) | 15.0 (4.8) | 0.032 | 0.085 |
| Dementia, N (%) | 5 (1.1) | 3 (3.0) | 17.0 (5.4) | 0.132 | 0.243 |
| Respiratory disease, N (%) | 106 (23.9) | 33 (33.0) | 77.2 (24.7) | 0.202 | 0.017 |
| Primary biliary cirrhosis (K74.3), N (%) | 443 (100) | 100 (100) | 312.8 (100) | <0.001 | <0.001 |
| Cirrhosis of liver (K74.6), N (%) | 46 (10.4) | 20 (20.0) | 32.5 (10.4) | 0.27 | <0.001 |
| Cholangitis (K83.0), N (%) | 43 (9.7) | 13 (13.0) | 16.7 (5.3) | 0.104 | 0.166 |
| Autoimmune hepatitis (K75.4), N (%) | 55 (12.4) | 9 (9.0) | 25.4 (8.1) | 0.111 | 0.142 |
| Obstruction of bile duct (K83.1), N (%) | 5 (1.1) | 8 (8.0) | 7.4 (2.4) | 0.334 | 0.094 |
| Hepatic failure (K72), N (%) | 16 (3.6) | 6 (6.0) | 3.3 (1.1) | 0.112 | 0.169 |
| Calculus of gallbladder  without cholecystitis (K80.2), N (%) | 16 (3.6) | 2 (2.0) | 3.7 (1.2) | 0.098 | 0.158 |
| Calculus of bile duct with  cholangitis (K80.3), N (%) | 3 (0.7) | 2 (2.0) | 9.2 (3.0) | 0.115 | 0.171 |
| Calculus of bile duct without cholangitis  or cholecystitis (K80.5), N (%) | 3 (0.7) | 0 (0.0) | 0.0 (0.0) | 0.117 | 0.117 |
| Liver transplantation, N (%) | 18 (4.1) | 19 (19.0) | 13.7 (4.4) | 0.481 | 0.015 |

*SMD: standardized mean difference, between exposed group and non-exposed group.


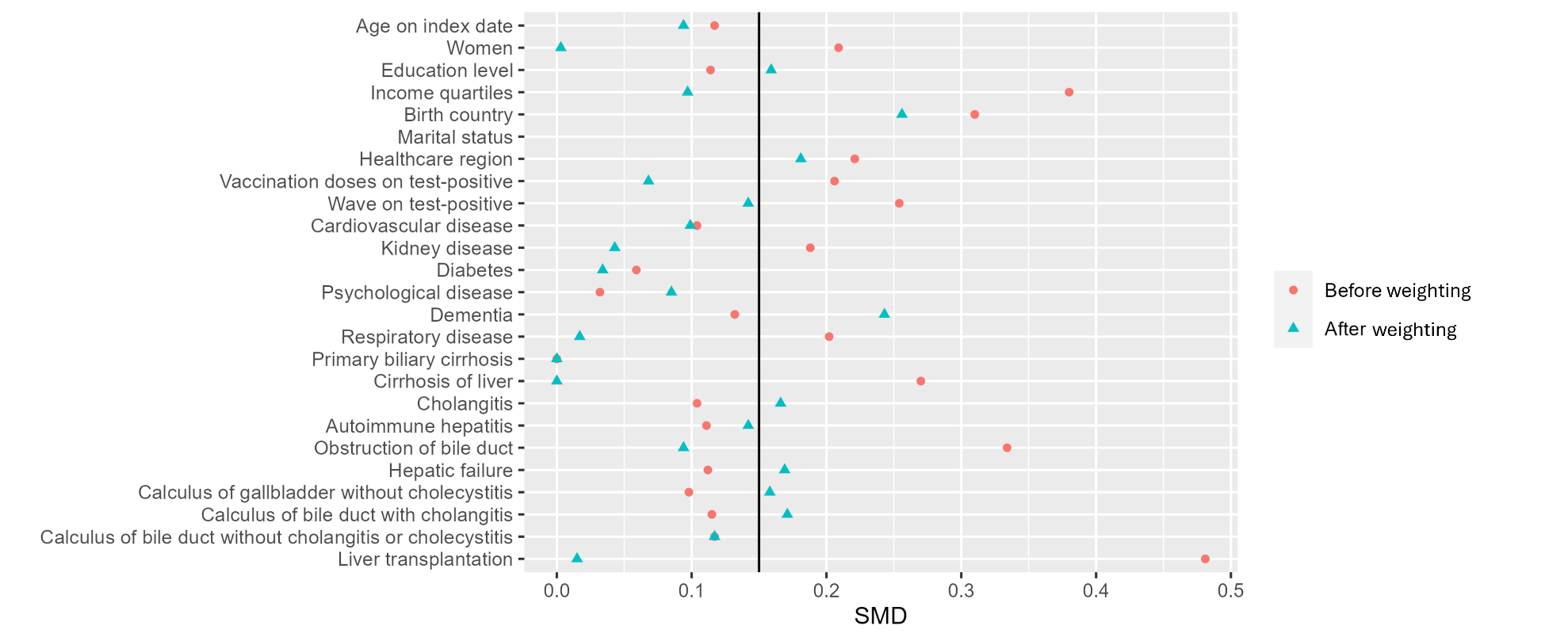


Figure S2. Standardized mean differences (SMD) in covariates before and after propensity score weighting in the ursodeoxycholic acid (UDCA) exposed and non-exposed groups in the PBC (primary biliary cirrhosis) cohort, among patients with a positive COVID-19 test in the Swedish population 1 Jan 2020 to 31 Dec 202 3. The PBC cohort consisted of all COVID-19 test-positive adults who had received a diagnosis of primary biliary cirrhosis (PBC, International Classification of Diseases, 10th revision (ICD-10) code K74.3), from 1 Jan 2015 to the date of each individual’s first positive test for SARS-CoV-2. The vertical black line represents SMD=0.15, values below it (dots on the left side of the line) represents good balance between groups.

Table S3. Demographic and socioeconomic characteristics and comorbidities of the ursodeoxycholic acid (UDCA) exposed and non-exposed group in the liver transplant cohort, among patients with a positive COVID-19 test in the Swedish population 1 Jan 2020 to 31 Dec 2023.

| Liver transplantation cohort | Exposed | Non-Exposed | | SMD* | |
| --- | --- | --- | --- | --- | --- |
|  |  | Before weighting | After weighting | Before weighting | After weighting |
| Count, N | 114 | 397 | 48.72 |  |  |
| Age on test-positive, mean (SD) | 52.18 (15.70) | 54.89 (14.94) | 51.57 (15.16) | 0.177 | 0.04 |
| Women, N (%) | 51 (44.7) | 149 (37.5) | 17.4 (35.8) | 0.147 | 0.183 |
| Education level, N (%) |  |  |  | 0.188 | 0.158 |
| primary | 16 (14.0) | 62 (15.6) | 4.8 (9.9) |  |  |
| upper-secondary | 48 (42.1) | 197 (49.6) | 21.7 (44.6) |  |  |
| tertiary | 48 (42.1) | 132 (33.2) | 21.8 (44.7) |  |  |
| unknown | 2 (1.8) | 6 (1.5) | 0.4 (0.8) |  |  |
| Income quartiles, N (%) |  |  |  | 0.089 | 0.106 |
| low | 33 (28.9) | 117 (29.5) | 13.3 (27.3) |  |  |
| lower-middle | 24 (21.1) | 81 (20.4) | 8.7 (17.8) |  |  |
| upper-middle | 30 (26.3) | 97 (24.4) | 14.2 (29.1) |  |  |
| high | 27 (23.7) | 101 (25.4) | 12.5 (25.7) |  |  |
| unknown | 0 (0.0) | 1 (0.3) | 0.0 (0.0) |  |  |
| Birth country, N (%) |  |  |  | 0.285 | 0.287 |
| Sweden | 86 (75.4) | 286 (72.0) | 38.0 (78.0) |  |  |
| high income | 8 (7.0) | 28 (7.1) | 2.8 (5.7) |  |  |
| low income | 8 (7.0) | 20 (5.0) | 2.0 (4.1) |  |  |
| lower-middle income | 1 (0.9) | 20 (5.0) | 2.1 (4.3) |  |  |
| upper-middle income | 9 (7.9) | 40 (10.1) | 3.6 (7.5) |  |  |
| unknown | 2 (1.8) | 3 (0.8) | 0.2 (0.4) |  |  |
| Marital status, N (%) |  |  |  | 0.128 | 0.06 |
| married | 55 (48.2) | 212 (53.4) | 24.9 (51.1) |  |  |
| not-married | 59 (51.8) | 184 (46.3) | 23.8 (48.9) |  |  |
| unknown | 0 (0) | 1 (0.3) | 0.0 (0) |  |  |
| Healthcare region, N (%) |  |  |  | 0.515 | 0.176 |
| North | 6 (5.3) | 22 (5.5) | 2.7 (5.5) |  |  |
| Stockholm | 36 (31.6) | 101 (25.4) | 15.3 (31.4) |  |  |
| Southeast | 10 (8.8) | 36 (9.1) | 4.1 (8.5) |  |  |
| South | 10 (8.8) | 76 (19.1) | 6.2 (12.7) |  |  |
| Uppsala-Örebro | 34 (29.8) | 58 (14.6) | 11.7 (23.9) |  |  |
| West | 18 (15.8) | 103 (25.9) | 8.8 (18.1) |  |  |
| unknown | 0 (0) | 1 (0.3) | 0.0 (0) |  |  |
| Vaccination doses on test-positive, N (%) |  |  |  | 0.089 | 0.107 |
| 0 dose | 47 (41.2) | 164 (41.3) | 21.0 (43.1) |  |  |
| 1 dose | 6 (5.3) | 15 (3.8) | 1.9 (3.9) |  |  |
| 2 doses | 12 (10.5) | 37 (9.3) | 4.0 (8.2) |  |  |
| 3 doses or more | 49 (43.0) | 181 (45.6) | 21.8 (44.8) |  |  |
| Wave on test-positive, N (%) |  |  |  | 0.092 | 0.127 |
| Wave 1 (Jan 2020 – Jan 2021) | 39 (34.2) | 122 (30.7) | 16.3 (33.5) |  |  |
| Wave 2 (Feb 2021 – Jun 2021) | 11 (9.6) | 47 (11.8) | 5.3 (11.0) |  |  |
| Wave 3 (Jul 2021 – Dec 2021) | 10 (8.8) | 35 (8.8) | 5.9 (12.1) |  |  |
| Wave 4 (Jan 2022 – Jun 2022) | 54 (47.4) | 193 (48.6) | 21.1 (43.4) |  |  |
|  |  |  |  |  |  |
| Cardiovascular disease, N (%) | 67 (58.8) | 243 (61.2) | 27.4 (56.3) | 0.05 | 0.051 |
| Kidney disease, N (%) | 18 (15.8) | 104 (26.2) | 7.7 (15.9) | 0.258 | 0.002 |
| Diabetes, N (%) | 30 (26.3) | 120 (30.2) | 13.2 (27.1) | 0.087 | 0.018 |
| Psychological disease, N (%) | 7 (6.1) | 34 (8.6) | 3.2 (6.5) | 0.093 | 0.014 |
| Dementia, N (%) | 0 (0.0) | 3 (0.8) | 0.2 (0.4) | 0.123 | 0.095 |
| Respiratory disease, N (%) | 41 (36.0) | 151 (38.0) | 18.0 (36.9) | 0.043 | 0.019 |
| Primary biliary cirrhosis (K74.3), N (%) | 18 (15.8) | 19 (4.8) | 4.6 (9.4) | 0.368 | 0.195 |
| Cirrhosis of liver (K74.6), N (%) | 42 (36.8) | 123 (31.0) | 16.7 (34.3) | 0.124 | 0.052 |
| Cholangitis (K83.0), N (%) | 66 (57.9) | 109 (27.5) | 25.1 (51.4) | 0.647 | 0.13 |
| Autoimmune hepatitis (K75.4), N (%) | 11 (9.6) | 32 (8.1) | 4.6 (9.4) | 0.056 | 0.008 |
| Obstruction of bile duct (K83.1), N (%) | 40 (35.1) | 56 (14.1) | 15.1 (31.1) | 0.502 | 0.085 |
| Hepatic failure (K72), N (%) | 18 (15.8) | 82 (20.7) | 8.0 (16.4) | 0.126 | 0.018 |
| Calculus of gallbladder  without cholecystitis (K80.2), N (%) | 1 (0.9) | 8 (2.0) | 0.5 (1.1) | 0.095 | 0.023 |
| Calculus of bile duct with  cholangitis (K80.3), N (%) | 7 (6.1) | 8 (2.0) | 1.5 (3.2) | 0.21 | 0.142 |
| Calculus of bile duct without cholangitis  or cholecystitis (K80.5), N (%) | 8 (7.0) | 10 (2.5) | 2.5 (5.2) | 0.212 | 0.077 |
| Liver transplantation, N (%) | 114 (100) | 397 (100) | 48.7 (100) | <0.001 | <0.001 |

*SMD: standardized mean difference, between exposed group and non-exposed group.


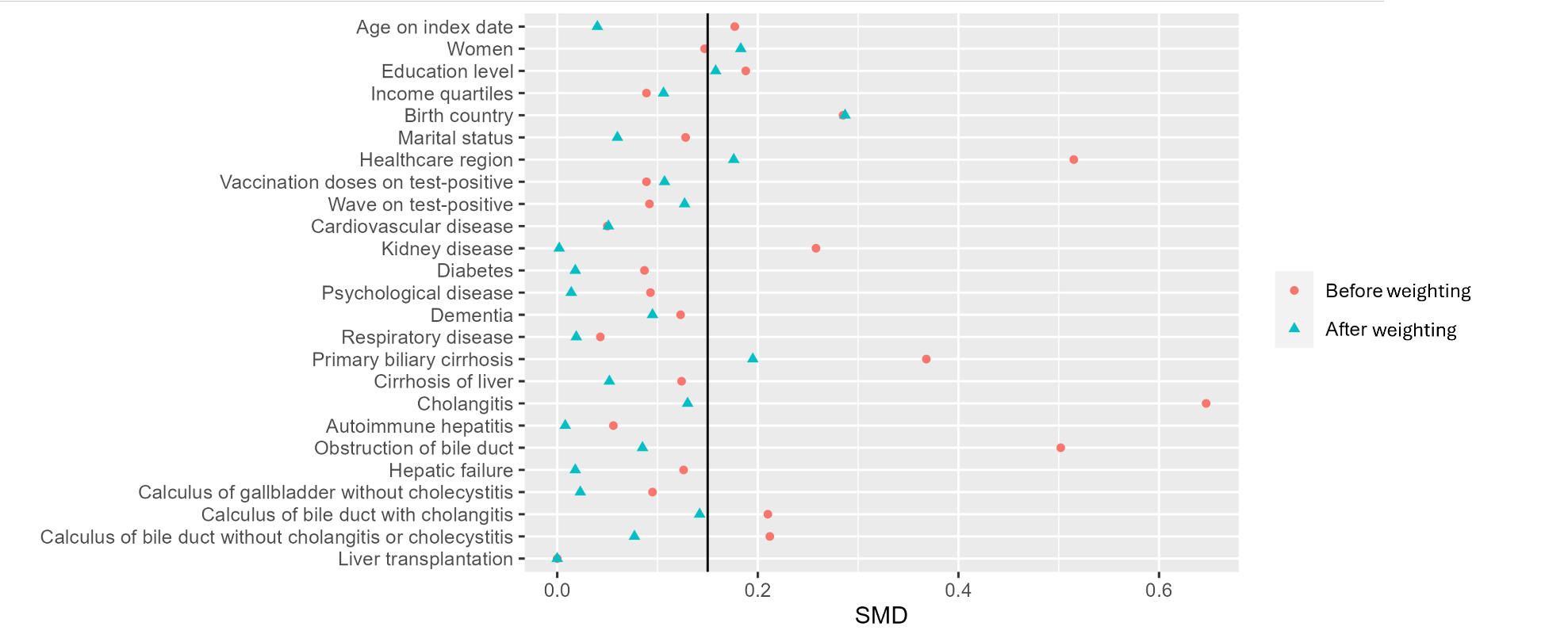


Figure S3. Standardized mean differences (SMD) in covariates before and after propensity score weighting in the ursodeoxycholic acid (UDCA) exposed and non-exposed groups in the liver transplantation cohort, among patients with a positive COVID-19 test in the Swedish population 1 Jan 2020 to 31 Dec 202 3. The liver transplantation cohort consisted of all COVID-19 test-positive adults had had a liver transplantation before and were still under immunosuppressor medication on the date of each individual’s first positive test for SARS-CoV-2. The vertical black line represents SMD=0.15, values below it (dots on the left side of the line) represents good balance between groups.


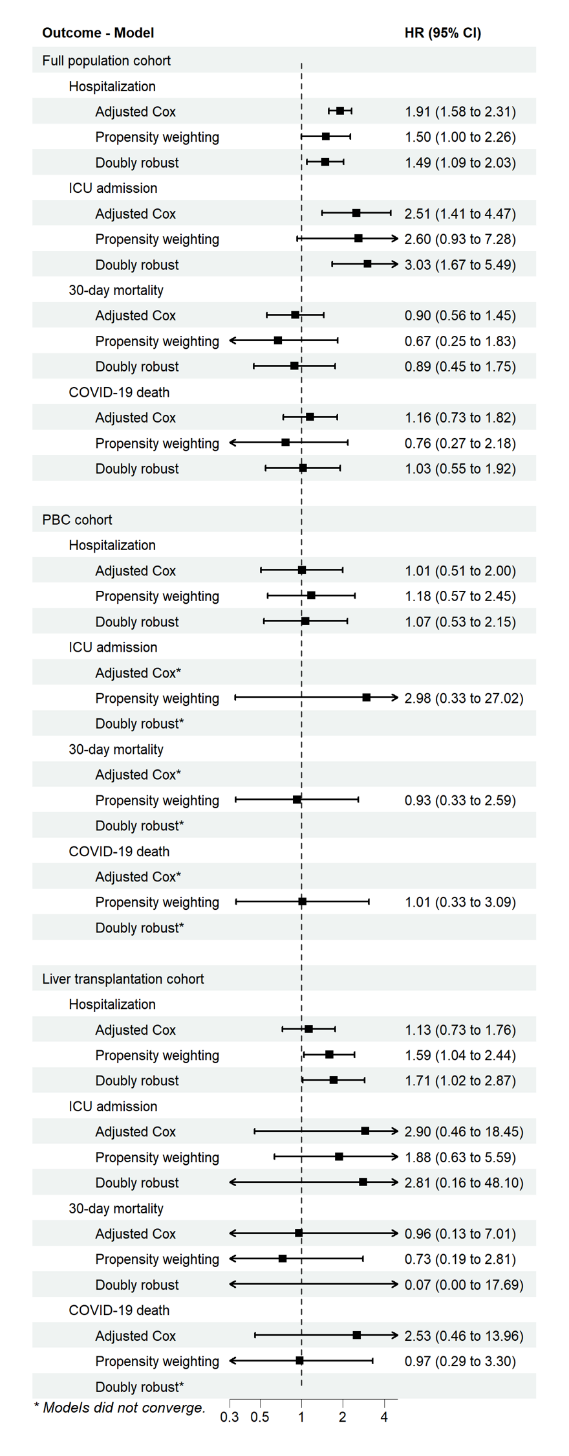


Figure S4. Hazard ratio and 95% confidence intervals of outcomes comparing ursodeoxycholic acid (UDCA) exposed to non-exposed group by different analytical methods in three different cohorts. The full population cohort consisted of all COVID-19 test-positive adults. The PBC cohort consisted of all COVID-19 test-positive adults who had received a diagnosis of primary biliary cirrhosis (PBC, International Classification of Diseases, 10th revision (ICD-10) code K74.3), from 1 Jan 2015 to the date of each individual’s first positive test for SARS-CoV-2. The liver transplant cohort consisted of all COVID-19 test-positive adults had had a liver transplantation before and were still under immunosuppressor medication on the date of each individual’s first positive test for SARS-CoV-2.
